# Hybrid CT-angiography to facilitate lower extremity sharp venous recanalization: a novel approach to a common procedure

**DOI:** 10.1186/s42155-020-00145-y

**Published:** 2020-10-08

**Authors:** Monica M. Matsumoto, Karan Nijhawan, Jeffrey A. Leef, Chelsea Dorsey, Osman Ahmed

**Affiliations:** 1 Department of Radiology, University of Pennsylvania, 3400 Spruce Street, Philadelphia, PA 19103 USA; 2 Department of Radiology, Section of Vascular and Interventional Radiology, University of Chicago Medicine, 5841 S. Maryland Ave, Chicago, IL 60637 USA; 3 Department of Surgery, Section of Vascular Surgery, University of Chicago Medicine, 5841 S. Maryland Ave, Chicago, IL 60637 USA

**Keywords:** Angio-CT, Sharp venous recanalization, Post-thrombotic syndrome, Iliocaval occlusion

## Abstract

**Background:**

Post-thrombotic syndrome due to chronic venous occlusion is associated with high morbidity and healthcare costs. Sharp venous recanalization has been used with success when conventional techniques fail to cross the occlusion, permitting endovascular reconstruction with angioplasty and stenting. However, manipulation of a needle, especially in extra-anatomic locations, risks damage to adjacent structures, thus necessitating adequate imaging guidance.

**Case presentation:**

This report describes the novel use of hybrid CT-angiography in a successful recanalization of a complex iliofemoral chronic venous occlusion, after multiple failed attempts with traditional recanalization techniques. The procedure was performed without complications, and stent patency was confirmed at three-month follow-up with patient-reported improvement in severe post-thrombotic syndrome.

**Conclusions:**

This case demonstrates effective incorporation of hybrid CT-angiography to facilitate complex sharp venous recanalization for chronic lower extremity thrombosis, as an alternative to standard fluoroscopic techniques requiring multiple projections with or without cone-beam CT. Further studies are needed to understand the implications of this strategy.

## Background

Post-thrombotic syndrome (PTS) is a common complication of deep vein thrombosis (DVT) and a major cause of morbidity and healthcare costs (Williams and Dillavou 2020). For PTS secondary to chronic iliocaval thrombosis in which standard endovascular reconstruction techniques fail to cross the occlusion, sharp recanalization has been reported with high technical success (McDevitt et al. 2019; Hage et al. 2018; Ito et al. 2012; Wadhwa et al. 2018). However, severe or moderate adverse events are reported in up to 5% of cases, so novel imaging methods beyond traditional fluoroscopic projection (with or without cone-beam computed tomography (CT)) that allow safer needle guidance would be clinically relevant (McDevitt et al. 2019; Ito et al. 2012; Wadhwa et al. 2018; Tanaka et al. 2014). We present a case of severe PTS, in which hybrid CT-angiography (angio-CT) permitted improved visualization for sharp recanalization and venous reconstruction of a complex chronic left common iliac vein (LCIV) occlusion.

## Case presentation

This case was exempt from the institutional review board. A 35-year-old male with a history of post-operative left lower extremity (LLE) DVT and recurrent right retroperitoneal Ewing sarcoma status post-chemotherapy, radiation, resection, and spinal reconstruction complicated by LCIV injury and repair, presented to our emergency department with severe left groin pain and LLE heaviness/edema. He had been discharged three days prior from an outside hospital, where he had undergone three rounds of catheter-directed thrombolysis for extensive acute on chronic LLE DVT, but multiple attempts to recanalize the LCIV occlusion from above and below using standard catheter and wire techniques had failed. He was on therapeutic enoxaparin and reported no chest or abdominal pain and no fever, cough, or chills. LLE duplex ultrasound demonstrated acute DVT with complete occlusion of the distal left external iliac (LEIV) and common femoral (LCFV) veins, partial occlusion of the left deep femoral vein, and acute-on-chronic DVT with partial occlusion of the proximal LCFV. On follow-up with vascular surgery, he received extensive counseling on treatment options and was referred to interventional radiology (IR) for endovascular recanalization; venous-venous bypass was offered as a second option. Physical exam demonstrated pain to palpation of the left groin, as well as edema, erythema, and tenderness of the left thigh and leg. Range of motion and pulses were intact. His Villalta score of 16 was compatible with severe PTS, and recanalization was attempted (Soosainathan et al. 2013).

**Fig. 1:**
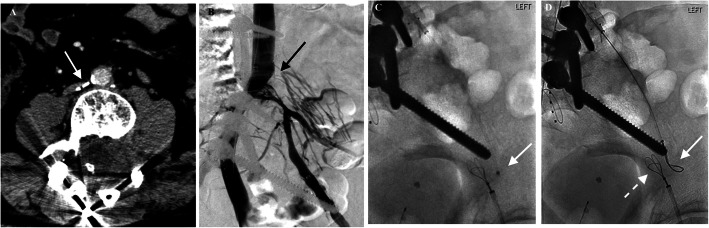
**a** CT demonstrated clips at level of LCIV origin (arrow) from prior surgical ligation of the LCIV, which were likely contributing to chronic occlusion of the vessel and complex recanalization techniques required; **b** Initial LLE venogram in the extreme RAO view shows flush occlusion of LCIV (arrow), in addition to postoperative findings related to L4 corpectomy and posterior lumbar fusion hardware; **c** Sharp recanalization needle was advanced from the proximal LCIV in the direction of the LEIV into the extra-vascular pelvic space (solid arrow), thus bypassing the known occlusion, confirmed on hybrid CT; **d** The two snares could now be advanced above (solid arrow) and below (dotted arrow) the area of occlusion and were confirmed to be close to each other on both AP and oblique projections

The initial LLE venogram confirmed the duplex ultrasound findings, and conventional recanalization attempts were unsuccessful (Fig. 1a), so the patient returned 1 month later for sharp recanalization under general anesthesia in a room equipped with angio-CT (Infinix-i 4DCT, Canon, Tustin, CA). Sheaths (Flexor; Cook Medical, Bloomington, IN) were placed in the right femoral vein (8F), LCFV (14F), and right internal jugular vein (RIJ) (8F) proximal to the iliac bifurcation. A 5F MPA catheter (Cook Medical) and 0.035″ hydrophilic guide wire (Glidewire; Terumo Interventional Systems, Somerset, NJ) could not be advanced further into the LCIV (Fig. 1b). The RIJ sheath was exchanged for a Rösch-Uchida transjugular liver access set (Cook Medical), EN Snare catheter systems (Merit Medical, South Jordan, UT) were placed in the RIJ and LCFV accesses, and the LCIV was recanalized with needle puncture across the surgical clips into the extra-vascular space under fluoroscopy (Fig. 1c-d).

**Fig. 2:**
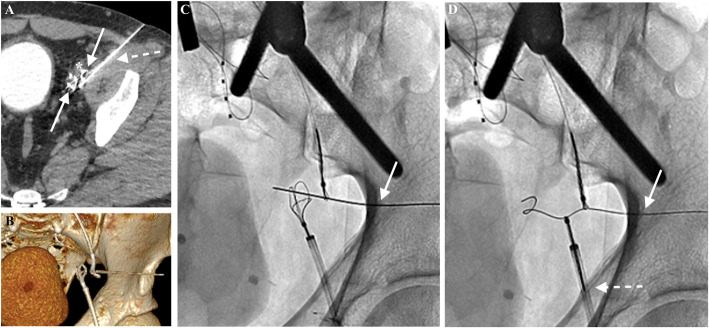
**a** Axial hybrid CT images confirmed the two snares (solid arrows) in close proximity to each other, with an adjacent external iliac artery (asterisk). A 21G needle (dotted arrow) was advanced percutaneously through the snare catheters using intermittent CT guidance, allowing a safe trajectory to avoid puncture of the artery and to reduce the potential for damage to adjacent structures; **b** Additional three-dimensional reformat show the trajectory of the needle (arrows) through the vascular snares; **c** Switching from CT to fluoroscopy allowed real-time adjustment and advancement of the needle (arrow) through both snares while avoiding the artery, utilizing the “gun-sight” approach without complication (Haskal et al. 1996); **d** A V-18 wire (solid arrow) was advanced through the needle access and snared through the LCFV access (dotted arrow). The needle was safely removed under fluoroscopy, and through-and-through “flossing” access was achieved with the following technique: the wire and snare were pulled out from the groin access, and a 0.018″ Quick-Cross catheter (Spectranetics, Colorado Springs, CO) was advanced over the wire through the superior snare and then snared from above with the wire removed. The wire was then re-advanced, snared, and pulled out from the neck access site

**Fig. 3:**
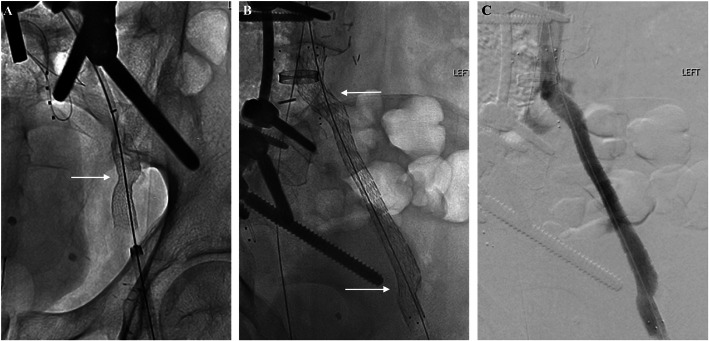
**a** Following balloon angioplasty of the LCIV and LEIV, a 13 mm Viabahn endoprosthesis (arrow) was deployed across the site of the extra-vascular connection created; **b** Additional Venovo stents (arrows) were deployed in the neo-LCIV and LEIV across the Viabahn with extension into the LCFV; **c** Post-angioplasty venogram showed patency of the left femoral and neo-iliac veins with restoration of normal luminal caliber

Hybrid-CT confirmed the extra-vascular location, as well as positioning of the snares (Fig. 2a). Under CT guidance, a 21G needle was advanced percutaneously through the snares. Under fluoroscopic guidance, an 0.018″ V-18 wire (Boston Scientific, Marlborough, MA) was advanced and snared through the LCFV access (Fig. 2a-c). Through-and-through “flossing” between the LCFV and RIJ accesses was achieved using a 0.035″ guidewire. Angioplasty (5x20mm Mustang; Boston Scientific) was performed over the LCIV and LEIV, followed by deployment of a Viabahn stent graft (13x50mm; Gore Medical, Flagstaff, AZ) across the extra-vascular connection site with post-dilation (12 × 40 mm Mustang) (Fig. 3a). A Viabahn stent was chosen to cross the extra-anatomic portion as it is self-expanding and can cover any potential area of injury at this point. Additional Venovo stents (16 × 160 mm, 12x60mm, 14x60mm) (BD Bard, Tempe, AZ) were deployed in the LCIV and LEIV across the Viabahn with extension into the LCFV, followed by angioplasty (14x40mm Atlas; BD Bard) (Fig. 3b). Rheolytic thrombectomy (AngioJet; Boston Scientific) was performed for acute thrombus throughout the LCFV and LEIV, with patency and restoration of normal luminal caliber on post-thrombectomy venogram (Fig. 3c).

The patient tolerated the procedure well without major complications. Serial creatinine remained at baseline, and hematocrit returned to normal after 24 h. The patient received a 300 mg loading dose of clopidogrel, in addition to heparin drip for 36 h, and was discharged on post-procedure day two on therapeutic enoxaparin and 75 mg clopidogrel daily. He reported marked clinical improvement in leg swelling at one-week follow-up phone call. On three-month follow-up he underwent IVC filter removal and LLE venogram, demonstrating widely-patent LCIV and LEIV stents. His Villalta improved to 6. He is currently scheduled for six-month venous duplex ultrasound follow-up.

## Conclusions

This case demonstrates strategic use of angio-CT to guide sharp recanalization of a chronic LCIV occlusion causing severe PTS in a patient with a complex surgical history and chronic occlusion refractory to prior recanalization attempts. Furthermore, long segment extra-anatomic sharp recanalization of the left iliocaval confluence left little room for technical error due to the proximity to the right iliac artery bifurcation (McDevitt et al. 2019; Ito et al. 2012). Given the need for precision and safety, angio-CT integrating volumetric CT fluoroscopy with fan beam CT machinery in the same room, as opposed to C-arm cone-beam CT, was utilized (Tanaka et al. 2014; Toyoda et al. 2009). The hybrid system is more frequently employed for interventional oncology and is not well described for chronic venous occlusions, but it assisted in completing this case efficiently and safely in the setting of spinal hardware and iliac artery proximity, with a larger axial field-of-view, improved real-time anatomic discrimination, quick interchanging between modalities, and artifact reduction with similar radiation doses to fan beam CT (Tanaka et al. 2014). Similar techniques would have been difficult with cone-beam CT, which requires either the C-arm to spin around the patient producing lower-quality images or fluoroscopy in multiple orthogonal planes. This case report demonstrates the utility and feasibility of incorporating angio-CT in complex sharp venous recanalization. Further studies are needed to understand the implications of this strategy.

## Data Availability

Data sharing is not applicable to this article as no datasets were generated or analysed during the current study.

## Abbreviations

Angio: Angiography
CT: Computed tomography
DVT: Deep venous thrombosis
IR: Interventional radiology
IVC: Inferior vena cava
LCFV: Left common femoral vein
LCIV: Left common iliac vein
LEIV: Left external iliac vein
LLE: Left lower extremity
PTS: Post thrombotic syndrome
RIJ: Right internal jugular vein
